# Differences in calcification and osteogenic potential of herniated discs according to the severity of degeneration based on Pfirrmann grade: a cross-sectional study

**DOI:** 10.1186/s12891-016-1015-x

**Published:** 2016-04-29

**Authors:** Jia Shao, Miao Yu, Liang Jiang, Feng Wei, Fengliang Wu, Zhongjun Liu, Xiaoguang Liu

**Affiliations:** Department of Orthopedics, Peking University Third Hospital, No. 49, North Garden Rd, Beijing, HaiDian District 100191 China

**Keywords:** Intervertebral disc herniation, calcified disc, osteogenic potential, Pfirrman grade, degeneration, Bone morphogenetic protein-2

## Abstract

**Background:**

Herniated discs may exhibit calcification, and calcified discs may complicate surgical treatment. However, the osteogenic potential and expression of osteogenic markers in degenerative discs of different degenerative grades are still unclear. Our purposes are to study the differences in calcification rate and osteogenic potential of herniated discs according to different degenerative grades.

**Methods:**

Fifty-eight lumbar intervertebral discs were removed from 41 patients. After grading according to the Pfirrmann scale, calcification was analyzed by micro computed tomography (μ-CT), and expression of osteogenic markers was analyzed by immunohistochemistry and real-time quantitative polymerase chain reaction (qPCR). Data from μ-CT scans were compared with the Kruskal–Wallis test. The Mann–Whitney test was applied to compare data between any two groups. Differences in osteogenic mRNA expression in different regions of the removed discs (posterior vs. anterior) were analyzed by paired *t* tests. Differences in the posterior portion of removed discs of different Pfirrmann grades were analyzed by one-way analysis of variance (ANOVA), and comparisons of data between discs of any two grades were completed with least significant difference (LSD) tests.

**Results:**

Significant differences in calcification according to μ-CT scanning were observed between discs of different degenerative grades. Nearly half of the discs of Pfirrmann grade V showed the highest degree of calcification compared to Pfirrman grade II discs. Bone morphogenetic protein (BMP)-2, Osterix, and Osteocalcin were detected histologically in discs of Pfirrmann grades III–V. Alkaline phosphatase (ALP) expression was observed in discs showing evidence of calcification. The qPCR analysis showed that BMP-2, Osterix, and Osteocalcin were expressed in most degenerated discs. We also observed greater expression of these osteogenic markers in the posterior portion of removed discs than in the anterior portion.

**Conclusions:**

The osteogenic potential of degenerated intervertebral discs appears to increase with the severity of degeneration and to be greater in the tissue near the spinal canal than in tissue in the inner portion of the disc.

## Background

Degenerative intervertebral disc disease leads to low back pain and radiating leg pain that severely diminishes patients’ quality of life [1]. Herniated discs may also exhibit calcification [2], and calcified discs may adhere to the epidural membrane, complicating surgical treatment and introducing a high risk of cerebral spinal fluid leakage [3]. Calcification of degenerative discs mainly resides in the herniated portion of the disc near the spinal canal. However, whether the osteogenic potential of the herniated intervertebral disc near the spinal canal is more powerful than the relative inner portion of the disc is still unclear. The expression of Bone morphogenetic protein 2 (BMP-2), which is widely known as an osteogenic induction factor, and its receptors in degenerated discs has been well elucidated [4], but the role of BMP-2 expression in intervertebral discs remains controversial [5–7]. BMP-2 has been shown to accelerate chondrogenesis and extracellular matrix formation [8, 9], and other studies have indicated that although BMP-2 cannot prevent intervertebral disc degeneration, it can lead to intervertebral disc ossification [7, 10]. Runx-2 is an essential transcription factor that initiates the hypertrophic differentiation of chondrocytes [11, 12], and Type X collagen expression is associated with endochondral ossification [13]. Overexpression of Runx-2 and Type X collagen has been observed in degenerated intervertebral discs, which indicates that degenerative discs have some extent of osteogenic potential [11, 14]. However, these reports did not consider the severity of degeneration or quantitatively compare the expression of osteogenic markers. Moreover, expression of some important osteogenic markers, including Osterix and Osteocalcin, has not been investigated in such discs.

The purpose of this study was to clarify the osteogenic potential and expression of osteogenic markers in degenerative discs of different Pfirrmann grades and to investigate the differences in osteogenic marker expression between the portion of the intervertebral disc near the spinal canal and that near the inner region of the disc.

## Methods

### Case selection

Forty-one cases were diagnosed as lumbar disc herniation or lumbar disc herniation combined with spinal stenosis and were treated by discectomy via a posterior approach in Peking University Third Hospital between February 2014 and December 2014. In 24 cases, a single segmental procedure was performed, whereas a two segmental procedure was performed in 17 cases. Fifty-eight discs were removed: 14 discs of L3/4, 31 of L4/5, and 13 of L5/S1. Patients with bone metabolic disease, congenital bone malformation, gout, renal dysfunction, or hypercalcemia were excluded. Our study was approved by the Medical Scientific Research Ethics Committee of Peking University Third Hospital, and the patients provided informed written consent to participation in this study.

### Reagents and antibodies

Total RNA was extracted using Trizol reagent (15596–026) from Invitrogen Life Technologies (Carlsbad, CS). Reverse transcription was performed using the RevertAid first strand cDNA synthesis kit (K1633) from ThermoScientific (Waltham, MA). The real-time quantitative polymerase chain reaction (qPCR) was carried out using Faststart Universal SYBR Green Master from Roche (Basel, Switzerland). Primers were synthesized by Invitrogen Biotechnology Co., Ltd., (Shanghai, China). Anti-BMP2 antibody (ab14933), anti-alkaline phosphatase (ALP) antibody (ab95462), anti-Sp7/Osterix antibody (ab94744), and anti-Osteocalcin antibody (ab93876) were purchased from Abcam (London, UK). Secondary antibody and a diaminobenzidine (DAB) kit (K5007) were obtained from DakoCytomation (Glostrup, Denmark).

### Grading of disc degeneration

The Pfirrmann grading system based on magnetic resonance imaging (MRI) T2 weighted images was used to classify the degenerative discs according to the severity of degeneration [15]. The operator and first assistant collaborated to judge the Pfirrmann grade of each disc, and the corresponding author made a final decision. Pfirrmann grade I indicates a normal, healthy disc as only found in children, whereas Pfirrmann grade V indicates the most severe degree of degeneration.

### Disc removal

After the lumbar lamina was removed, the nerve root was retracted in the posterior lumbar discectomy procedure, leaving space for the posterior longitudinal ligament and annulus fibrosus of the herniated side to be incised circularly. Then the discs were removed using the nucleus pulposus forceps. We designated the first piece of tissue removed as the specimen. Eighteen specimens (3 of grades II and V, 6 of grades III and IV) were selected to be fixed in test tubes filled with 10 % neutral buffered formalin with the anterior part of the specimen leaning on the bottom of the tube for 12–24 h. Specimens were then washed and temporarily steeped in 75 % ethanol at 4 °C before μ-CT analysis. The other forty samples (6 of grade II, 14 of grade III, 12 of grade IV, and 8 of grade V) were transferred to a frozen storage tube immediately with the anterior part of the specimen leaning on the bottom of the tube and stored at −80 °C until analysis.

### Micro computed tomography (μ-CT)

All 58 specimens were analyzed using a Siemens Inveon μ-CT scanner (Siemens Medical Solutions, Knoxville, TN) according to the following parameters: X-ray beam voltage, 80 kV; current, 500 μA; and effective resolution, 13.6 μm. According to the semi-quantitative grading system of Rutges et al. [16], we designated the absence of calcification as -, the presence of a single area of calcification as ±, the presence of two clear areas of calcification as +, and the clear presence of multiple areas of calcification as ++.

### Histological analysis

Hematoxylin-eosin (HE) staining was used to evaluate tissue organization, Von Kossa staining was used to determine mineralization, and immunohistochemical staining was applied to investigate the expression of BMP-2, Osterix, Osteocalcin, and ALP. After dehydration and paraffin embedding, the specimens were sectioned at 5 μm. The sections were deparaffinized and hydrated, and heat treatment in citrate buffer (pH 6.0) was used for antigen retrieval. After the primary antibodies were added at proper dilutions (1:400 for anti-BMP-2, 1:200 for anti-Osterix, 1:200 for anti-Osteocalcin, and 1:400 for anti-ALP), the sections were incubated for 2 hours at 37 °C. After addition of the secondary antibody, DAB was used as the chromogen. All sections were counterstained with hematoxylin.

### mRNA extraction and cDNA synthesis

After μ-CT analysis, each removed intervertebral disc was divided into posterior and anterior parts for qPCR analysis. After being mixed with Trizol reagent and ground sufficiently, the specimens were centrifuged and reconstituted in Methenyltrichloride and propyl alcohol. The product of centrifugation was total RNA. Reverse transcription for cDNA synthesis was completed following instructions provided by the manufacturer of the kit employed (RevertAid first strand cDNA synthesis kit).

### qPCR

β-actin was used as the reference gene. We randomly chose five posterior portions of samples from each grade, and eight samples that had been divided into posterior and anterior parts for qPCR analysis. Because no osteogenic markers were detected in grade II samples in our preliminary experiment, grade II samples were excluded from qPCR analysis. qPCR was completed following the protocol provided by the manufacturer of the product (Faststart Universal SYBR Green Master) with the primers listed in Table 1. The PCR program was set as follows: pre-denaturation at 95 °C for 10 min (1 cycle), 40 amplification cycles (95 °C for 15 s, cooling to 60 °C and holding for 60 s), and then an increase from 75 °C to 95 °C at 1 °C per 20 seconds to obtain the melting curve. Relative gene expression was normalized to that in the anterior portion of the removed discs when samples were grouped according to the removed part of the disc, and by Pfirrmann Grade III when grouped by Pfirrmann grade through application of the Livak method [17].

**Table 1:** Primers for real-time PCR

Gene	Upstream primer	Downstream primer
β-actin	CACCCAGCACAATGAAGATCAAGAT	CCAGTTTTTAAATCCTGAGTCAAGC
BMP-2	CTGAACTCCACTAATCATGCCAT	ACCCACAACCCTCCACAACC
SP7 (Osterix)	TCTGCGGGACTCAACAACTCT	TGGGAAAAGGGAGGGTAATCA
BGLAP (Osteocalcin)	AGGGCAGCGAGGTAGTGAAG	TCCTGAAAGCCGATGTGGTC

### Statistical analysis

Statistical analysis was completed using SPSS 20.0 for Windows PC version (SPSS Inc., Chicago, IL). The kappa value was calculated to determine interobserver agreement regarding the Pfirrmann grade. Data from μ-CT scans were compared with the Kruskal–Wallis test. The Mann–Whitney test was applied to compare data between any two groups. Differences in osteogenic mRNA expression in different regions of the removed discs (posterior vs. anterior) were analyzed by paired *t* tests. Differences in the posterior portion of removed intervertebral discs of different Pfirrmann grades were analyzed by one-way analysis of variance (ANOVA), and comparisons of data between discs of any two grades were completed with least squares difference (LSD) tests. *P* < 0.05 was considered statistically significant, except in the Mann–Whitney test for which a corrected *P* < 0.007 as statistically significant. The corrected *P* value in the Mann–Whitney test was calculated using the following formula:

$$ \mathrm{P}\hbox{'}=\frac{\mathrm{P}}{{\mathrm{C}}_{\mathrm{n}}^2+1} $$

where P’ indicates the corrected *P* value (*P* = 0.05), and “n” represents the number of groups, which was 4 in our study. P’ was approximately 0.0071 based on the formula, and thus, we defined P’ < 0.007 as statistically significant.

## Results

**Fig. 1:**
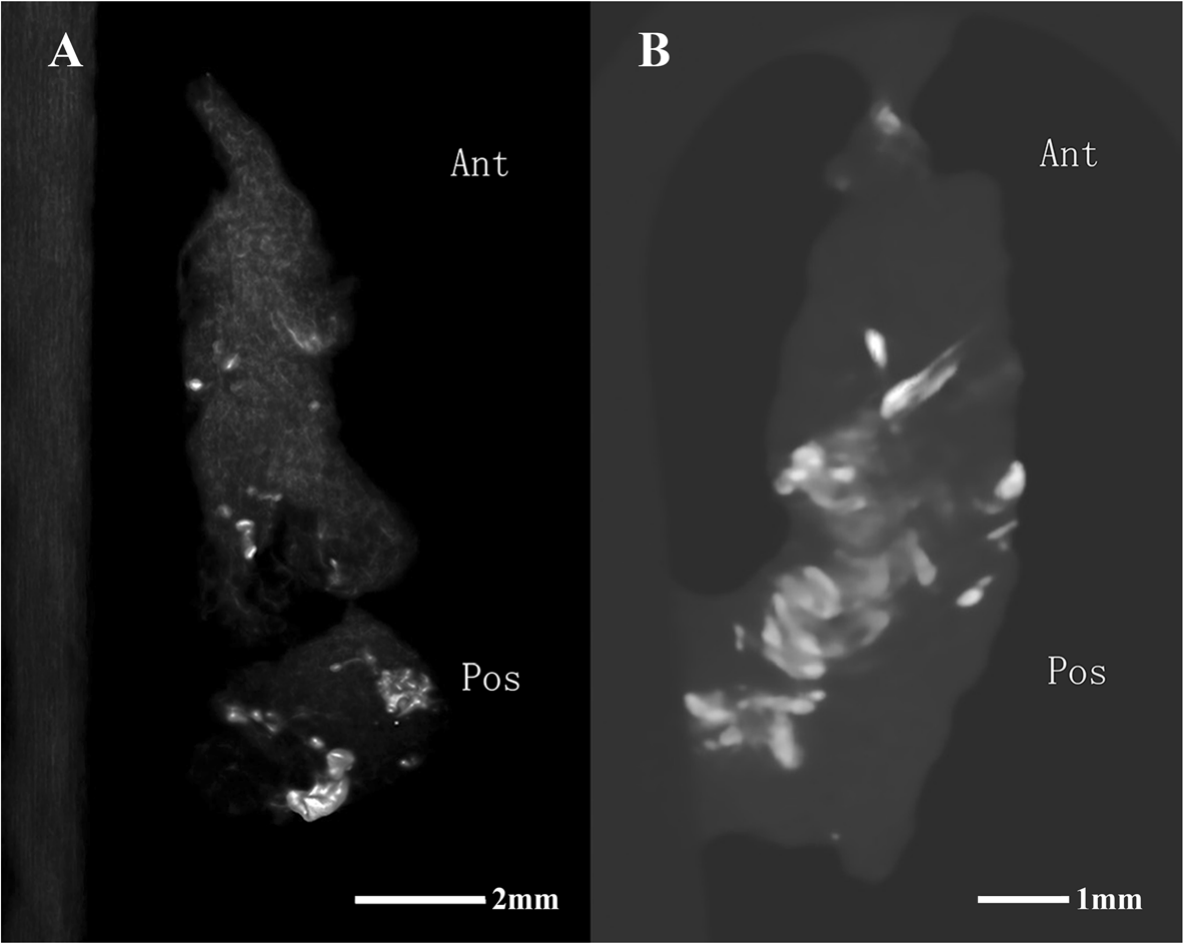
μ-CT scan revealing that the posterior portions of the removed intervertebral discs contained more ossification sites than the anterior portions. White spots indicate the calcification spots in the removed disc. **a** is a Pfirrmann grade III disc, and (**b**) is a Pfirrmann grade V disc

The interobserver kappa value for determining the Pfirrmann grade was 0.792 (*P* < 0.01). The μ-CT results for intervertebral disc calcification in discs of different Pfirrmann grades indicated that the percentage of discs with a calcification grade of ++ increased from 0 % among the Pfirrman grade II discs to 45.4 % among the Pfirrmann grade V discs (Table 2). The corresponding Kruskal–Wallis test results were χ^2^ = 19.274 with a *P* < 0.001, and the Mann–Whitney test results showed a significant difference between grades II and III (*P* < 0.007), grades II and IV (*P* < 0.001), and grades II and V (*P* < 0.001), whereas no significant difference in grades III and IV (*P* = 0.105), grades III and V (*P* = 0.174), and grades IV and V (*P* = 0.805) was observed. Thus, our μ-CT results indicate a significant difference in the extent of degeneration and calcification between discs of Pfirrmann grade II vs. III, IV, or V. In addition, we observed more ossification sites in the posterior portions of removed intervertebral discs than in the anterior portions (Fig. 1).

**Table 2:** Calcification rate based on μ-CT

N = 58	No. of cases	-	±	+	++
Pfirrman II	9	6 (66.7 %)	3 (33.3 %)	0 (0 %)	0 (0 %)
Pfirrman III	20	4 (20 %)	4 (20 %)	7 (35 %)	5 (25 %)
Pfirrman IV	18	0 (0 %)	2 (11.1 %)	10 (55.6 %)	6 (33.3 %)
Pfirrman V	11	1 (9.1 %)	1 (9.1 %)	4 (36.4 %)	5 (45.4 %)

**Fig. 2:**
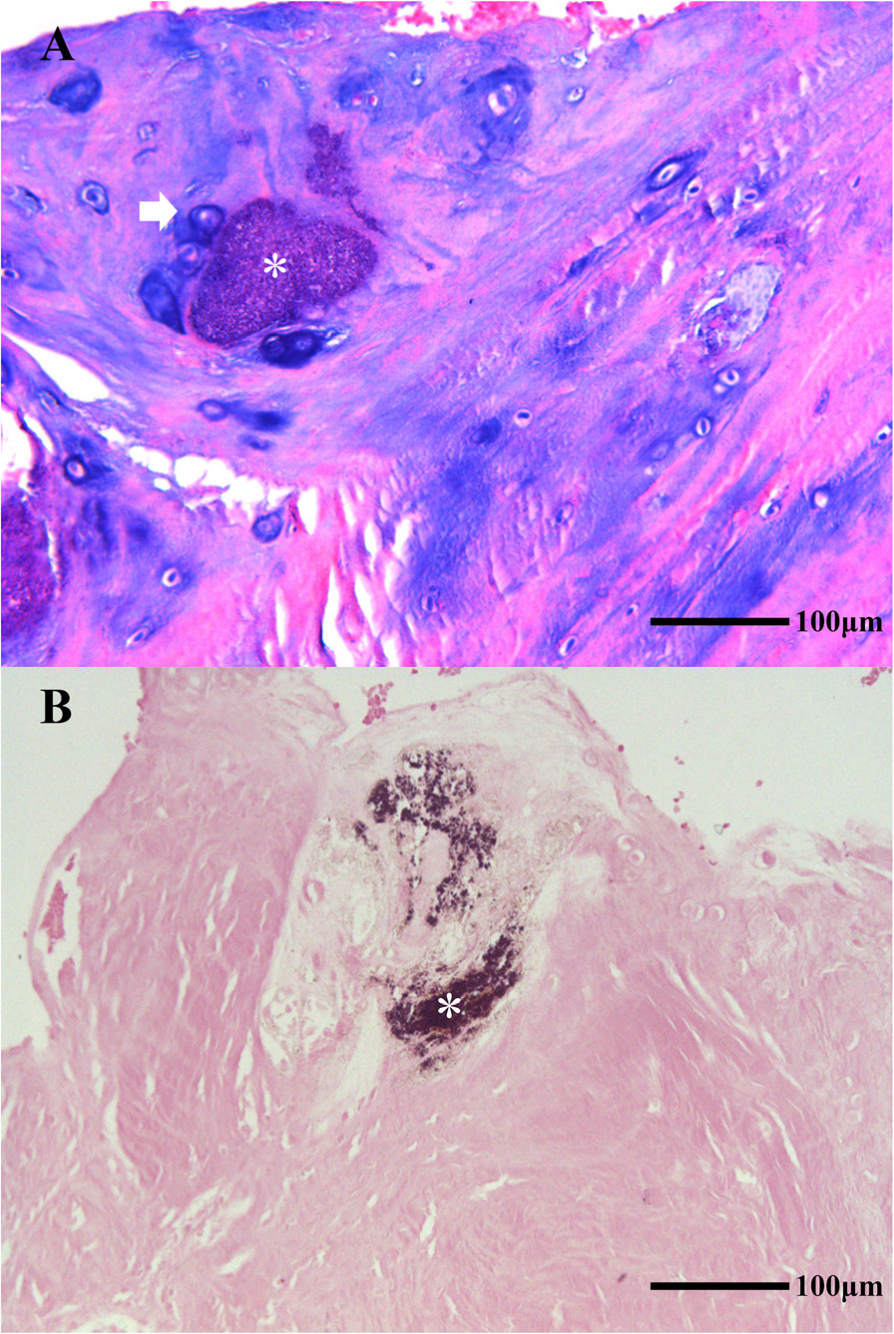
HE staining in (**a**) revealed mineralization (*) and cells surrounding the mineralization spot (arrow) in degenerated discs. Von Kossa staining in (**b**) verified the mineralization (*) in discs. **a** and **b** are Pfirrmann grade IV discs

**Fig. 3:**
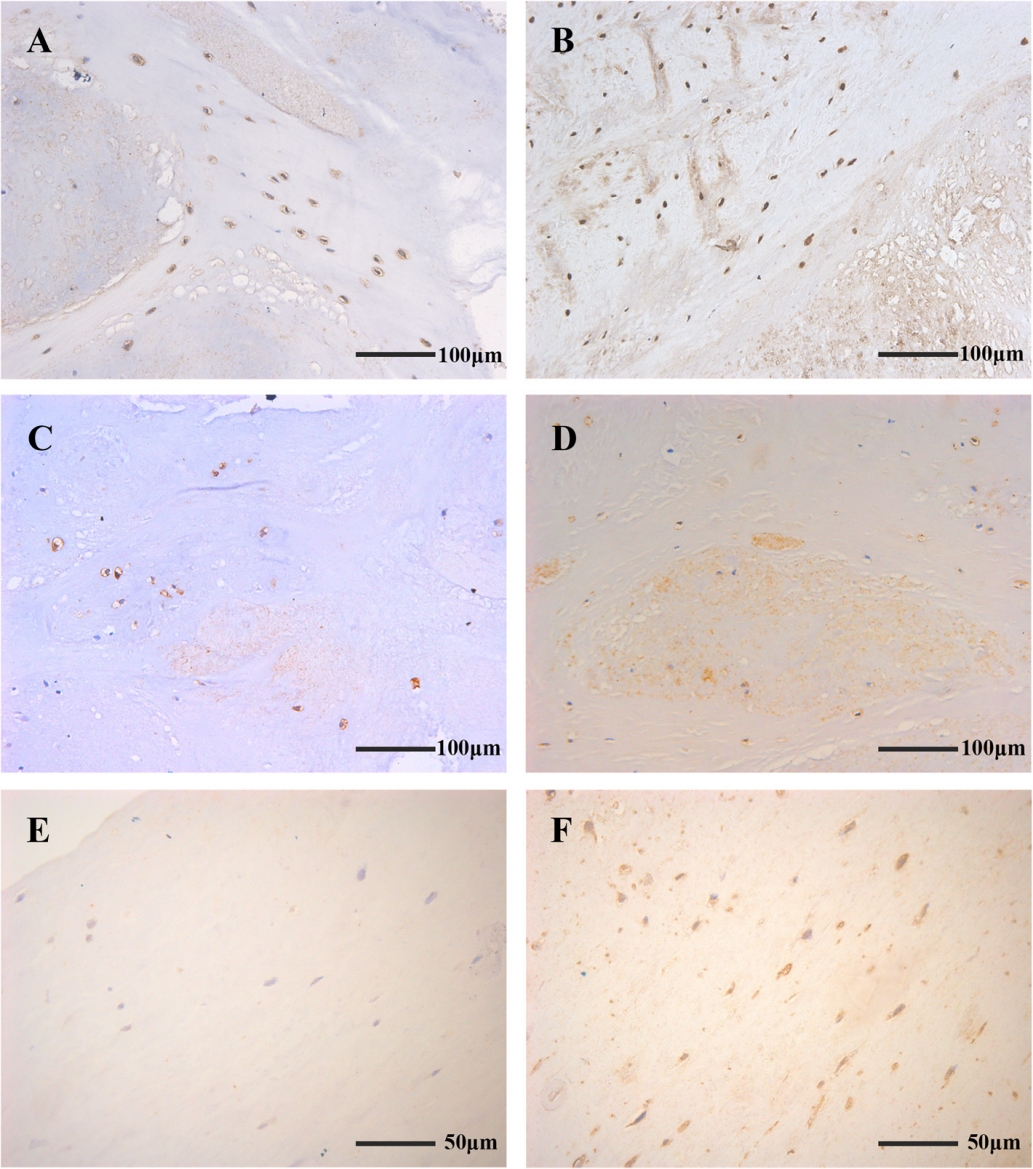
BMP-2 (**a**), Osterix (**b**), and Osteocalcin (**c**) were detected in degenerated discs, even if μ-CT did not show calcification. ALP (**d**) was positive in herniated discs with evidence of calcification. In discs for which μ-CT indicated – or ± calcification, ALP expression was not detected or detected in only a few cells (**e**), whereas staining for Osterix (**f**) was positive in the same position. **a** and **d** are Pfirrmann grade IV discs, **b** and **c** belong to grade V, and **e** and **f** are grade III

Mineralization was detected in degenerated discs by HE staining, especially in the specimens that exhibited calcification on μ-CT (Fig. 2a), in which more cells were detected surrounding the calcification site. Von Kossa staining verified the mineralization of spots as shown in Fig. 2b. Moreover, based on immunohistochemical staining, discs of Pfirrman grades III, IV, and V showed expression of BMP-2, Osterix, and Osteocalcin (Fig. 3a, b and c), even if the μ-CT results did not reveal calcification. ALP expression was observed only in the specimens for which μ-CT showed calcification categorized as + or ++ (Fig. 3d). In specimens with – or ± calcification scoring, ALP expression was not detected or detected in only a few cells (Fig. 3e), whereas staining for Osterix (Fig. 3f) was positive.

**Fig. 4:**
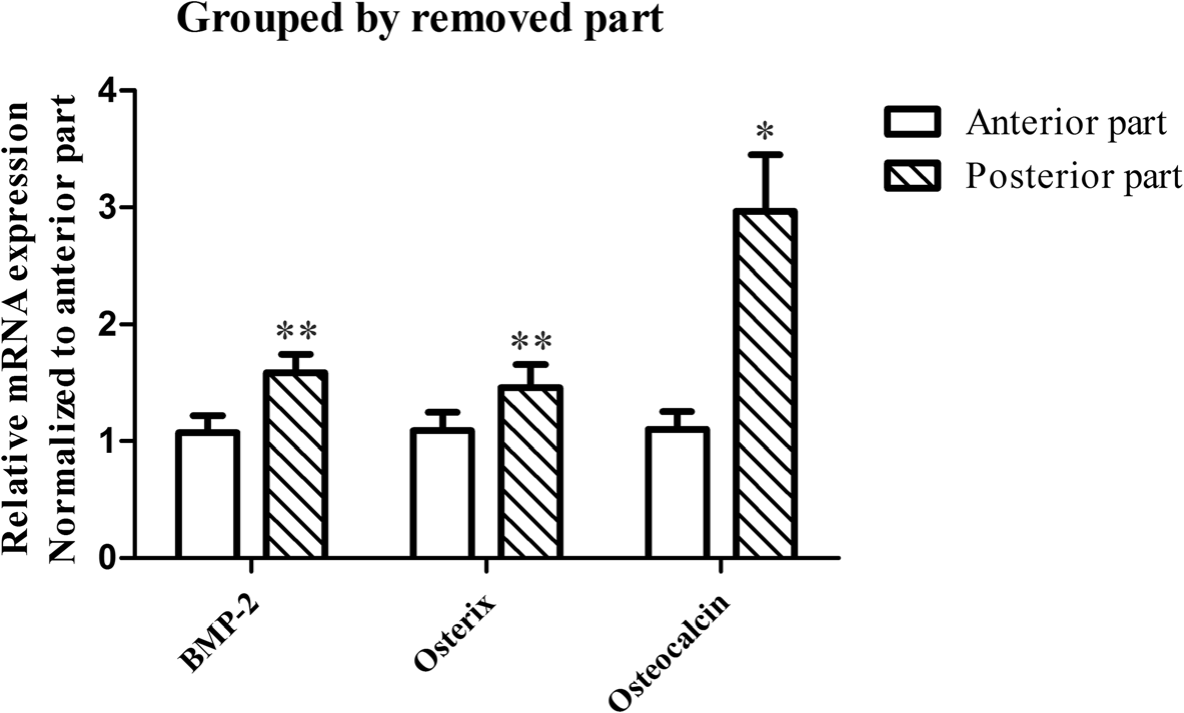
BMP-2, Osterix, and Osteocalcin expression was greater in the posterior part of the removed intervertebral disc than in the anterior part. *P < 0.05, **P < 0.01

The qPCR analysis revealed that most degenerated discs, except those of Pfirrmann grade II and two specimens of Pfirrmann grade III, expressed osteogenic markers. In addition, according to comparisons by paired t tests, the posterior portions of removed discs showed greater expression of BMP-2 (*P* < 0.01), Osterix (*P* < 0.01), and Osteocalcin (*P* < 0.05) than the anterior portions (Fig. 4).

**Fig. 5:**
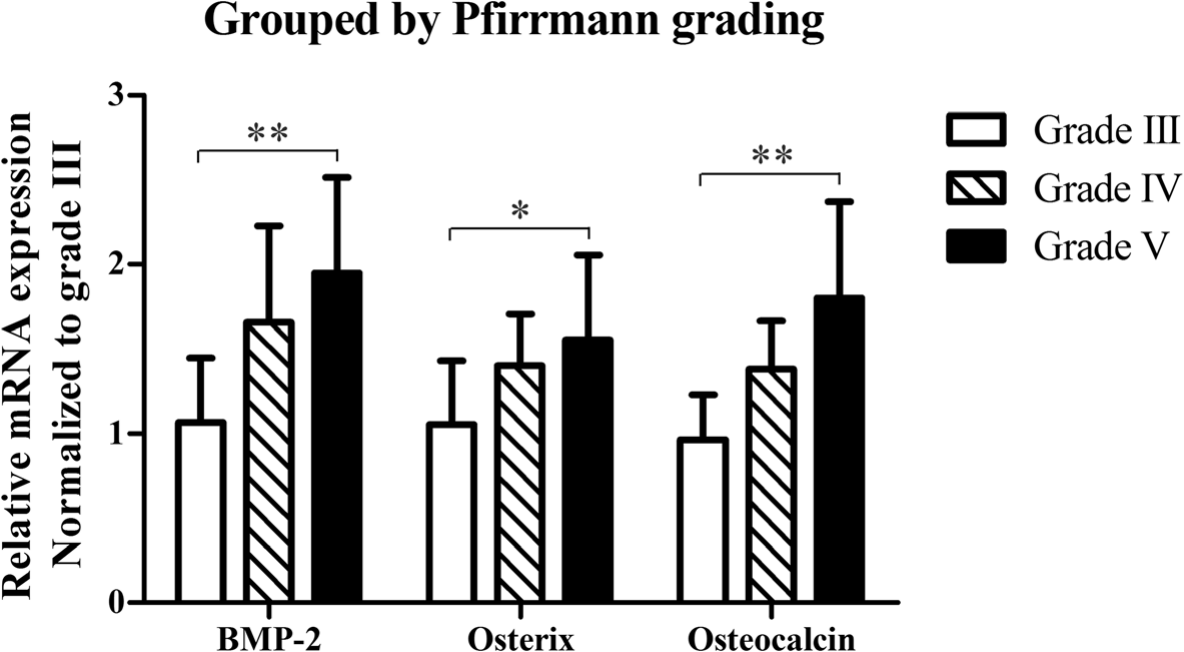
Greater expression of BMP-2 (*P* < 0.01), Osterix (*P* < 0.05), and Osteocalcin, (*P* < 0.01) was observed in Pfirrmann grade V discs compared to grade III discs. *P < 0.05, **P < 0.01

One-way ANOVA comparing osteogenic marker expression among discs of different Pfirrrmann grades also confirmed significant differences in the expression of BMP-2 (*P* < 0.01) and the Osteocalcin group (*P* < 0.05), but no differences were observed in Osterix expression (*P* = 0.124). Although LSD tests revealed no statistically significant differences in gene expression between any pair of adjacent Pfirrmann grades, the comparison of gene expression data for Pfirrmann Grade III and Grade V discs indicated that grade V discs expressed higher levels of BMP-2 (*P* < 0.01), Osterix (*P* < 0.05), and Osteocalcin (*P* < 0.01) than the grade III group (Fig. 5).

## Discussion

Our μ-CT results indicate a significant increase in the degree of intervertebral disc calcification with increasing severity of degeneration based on the Pfirrmann grading system, and this finding is consistent with the results of a previous study [18]. The lack of significant differences in calcification between discs of Pfirrmann grades III–V may have been due to the use of calcification grading system that is semi-quantitative, focusing only on the number of calcified spots and not considering the size of such regions. There was one specimen of Pfirrmann grade V that appeared to have a “-” grade of calcification. Although no calcification spot may have formed in this sample or the formed calcification was below the resolution of μ-CT, positive Osterix expression on immunohistochemical staining indicated the osteogenic potential of the disc. Our μ-CT results also showed that the posterior part of the removed intervertebral discs showed much greater calcification than the anterior part.

Dystrophic calcification is a passive pathologic process caused by necrosis or malnutrition, but it has been reported that the calcification of intervertebral discs is an active process that occurs along with hypertrophic differentiation [16]. Our observation of cells expressing BMP-2, Osterix, Osteocalcin, and ALP around and/or within calcified tissue as well as the increased gene expression of BMP-2, Osterix, and Osteocalcin in discs exhibiting greater calcification indicate that intervertebral disc calcification is an active process linked to osteogenesis. Moreover, based on the expression of Runx-2 in degenerated discs [11], the ossification of intervertebral discs is an endochondral process.

BMP-2 is a type of multifunctional growth factor involved in the regulation and control of development, differentiation, and apoptosis of osteoblasts, chondroblasts, neurocytes, and epithelial cells [19]. Takae et al. [4] first reported the expression of BMP-2 and its receptors in degenerated cervical intervertebral discs of mice in 1999. Although their results indicated a relationship between BMP-2 expression and disc degeneration, the exact relationship remained unclear. A series of in vitro and in vivo studies has elucidated the roles of chondrogenesis and the regenerative effect of BMP-2 in the intervertebral disc degeneration process [20, 21]. However, another study [7] demonstrated Osteocalcin expression and calcium deposition after treatment of intervertebral disc cells with BMP-2, indicating that BMP-2 has an osteogenic effect in intervertebral disc tissue. Our findings indicate that the level of BMP-2 expression in intervertebral disc tissue is related to the severity of degeneration, in accordance with the results of a previous study [22]. Our data also reveal that the expression levels of Osterix and Osteocalcin follow trends similar to BMP-2, confirming that the BMP-2 level is consistent with the osteogenic potential of degenerated discs. We hypothesize that BMP-2 plays two roles in degenerated discs: one is compensatory in which BMP-2 accelerates cartilage repair, and because this compensatory expression cannot completely reverse the degeneration, overexpression of BMP-2 finally leads to the ossification of discs as a side effect. The underlying mechanisms by which BMP-2 regulates chondrogenesis and osteogenesis remain unknown, and in our future studies, we will investigate methods to block the osteogenic effect of BMP-2 to ensure proper intervertebral disc regeneration upon treatment with BMP-2.

Our μ-CT and qPCR results both demonstrated that the posterior part of removed intervertebral discs possesses greater osteogenic potential than the anterior part. This phenomenon may be caused by differences in the local environments of these regions of discs. Interestingly, the posterior part of intervertebral discs has also been shown to exhibit more severe inflammation, fibrosis, and angiogenesis than the anterior part [18]. However, the presence of some degree of osteogenic potential in the anterior part indicates that the differences in the local environment are not the only cause for this phenomenon, and some other undefined factors likely participate in the induction of intervertebral disc ossification.

Our immunohistochemical staining experiments revealed the presence of the osteogenic markers BMP-2, Osterix, Osteocalcin, and especially ALP, which hydrolyzes calcium pyrophosphate [23] to initiate an irreversible process of calcification, and even in discs that did not stain positively for ALP, Osterix expression was observed. These findings suggest that osteogenesis in the intervertebral disc is a very slow process first involving the formation of dispersed, amorphous regions of calcification or osteoid tissue with eventual formation of mature bone in the long term. The formation of these calcified tissues may be related to the inflammation and angiogenesis that occur after disc degeneration or herniation. The origin of osteoprogenitor cells responsible for intervertebral disc ossification is still not clear. These cells may be derived from primitive cells in the peripheral blood and/or from osteoprogenitor cells in the degenerated disc [24]. Jin et al. reported that the origin of these cells is the pre-existing primitive cells in the disc. However, other studies have shown that the cells responsible for ossification in aortic calcification and tendinopathy are derived from the two different origins, which suggests this possibility also in intervertebral discs [25, 26].

## Conclusions

Degenerated intervertebral discs appear to possess osteogenic potential in direct relation to the severity of degeneration. The more severely degenerated discs have greater osteogenic potential than the less severely degenerated discs. Moreover, the osteogenic potential of the intervertebral disc tissue near the spinal canal is greater than that of the tissue in the relative inner portion of the disc.

### Ethics approval and consent to participate

Our study was approved by the Medical Scientific Research Ethics Committee of Peking University Third Hospital, and the patients provided informed written consent to participation in this study.

### Consent for publication

Not applicable.

### Availability of data and materials

The datasets supporting the conclusions of this article are included within the article.

## Abbreviations

μ-CT: Micro computed tomography
qPCR: Quantitative polymerase chain reaction
ANOVA: Analysis of variance
LSD: Least significant difference
BMP: Bone morphogenetic protein
ALP: Alkaline phosphatase
MRI: Magnetic resonance imaging
HE: Hematoxylin-eosin

## References

[1] Luoma K, Riihimaki H, Luukkonen R, Raininko R, Viikari-Juntura E, Lamminen A (2000). Low back pain in relation to lumbar disc degeneration. Spine (Phila Pa 1976).

[2] Urban JP, Roberts S (2003). Degeneration of the intervertebral disc. Arthritis Res Ther..

[3] Barbanera A, Serchi E, Fiorenza V, Nina P, Andreoli A (2009). Giant calcified thoracic herniated disc: considerations aiming a proper surgical strategy. J Neurosurg Sci..

[4] Takae R, Matsunaga S, Origuchi N, Yamamoto T, Morimoto N, Suzuki S (1999). Immunolocalization of bone morphogenetic protein and its receptors in degeneration of intervertebral disc. Spine (Phila Pa 1976).

[5] Kim DJ, Moon SH, Kim H, Kwon UH, Park MS, Han KJ (2003). Bone morphogenetic protein-2 facilitates expression of chondrogenic, not osteogenic, phenotype of human intervertebral disc cells. Spine (Phila Pa 1976).

[6] Kim H, Lee JU, Moon SH, Kim HC, Kwon UH, Seol NH (2009). Zonal responsiveness of the human intervertebral disc to bone morphogenetic protein-2. Spine (Phila Pa 1976).

[7] Haschtmann D, Ferguson SJ, Stoyanov JV (2012). BMP-2 and TGF-beta3 do not prevent spontaneous degeneration in rabbit disc explants but induce ossification of the annulus fibrosus. Eur Spine J..

[8] Li J, Kim KS, Park JS, Elmer WA, Hutton WC, Yoon ST (2003). BMP-2 and CDMP-2: stimulation of chondrocyte production of proteoglycan. J Orthop Sci..

[9] Tim Yoon S, Su Kim K, Li J, Soo Park J, Akamaru T, Elmer WA (2003). The effect of bone morphogenetic protein-2 on rat intervertebral disc cells in vitro. Spine (Phila Pa 1976).

[10] Wang Z, Hutton WC, Yoon ST (2013). ISSLS Prize winner: Effect of link protein peptide on human intervertebral disc cells. Spine (Phila Pa 1976).

[11] Sato S, Kimura A, Ozdemir J, Asou Y, Miyazaki M, Jinno T (2008). The distinct role of the Runx proteins in chondrocyte differentiation and intervertebral disc degeneration: findings in murine models and in human disease. Arthritis Rheum..

[12] Takeda S, Bonnamy JP, Owen MJ, Ducy P, Karsenty G (2001). Continuous expression of Cbfa1 in nonhypertrophic chondrocytes uncovers its ability to induce hypertrophic chondrocyte differentiation and partially rescues Cbfa1-deficient mice. Genes Dev..

[13] Shen G (2005). The role of type X collagen in facilitating and regulating endochondral ossification of articular cartilage. Orthod Craniofac Res..

[14] Hristova GI, Jarzem P, Ouellet JA, Roughley PJ, Epure LM, Antoniou J (2011). Calcification in human intervertebral disc degeneration and scoliosis. J Orthop Res..

[15] Pfirrmann CW, Metzdorf A, Zanetti M, Hodler J, Boos N (2001). Magnetic resonance classification of lumbar intervertebral disc degeneration. Spine (Phila Pa 1976).

[16] Rutges JP, Duit RA, Kummer JA, Oner FC, van Rijen MH, Verbout AJ (2010). Hypertrophic differentiation and calcification during intervertebral disc degeneration. Osteoarthritis Cartilage..

[17] Livak KJ, Schmittgen TD (2001). Analysis of relative gene expression data using real-time quantitative PCR and the 2(−Delta Delta C(T)) Method. Methods..

[18] Karamouzian S, Eskandary H, Faramarzee M, Saba M, Safizade H, Ghadipasha M (2010). Frequency of lumbar intervertebral disc calcification and angiogenesis, and their correlation with clinical, surgical, and magnetic resonance imaging findings. Spine (Phila Pa 1976).

[19] Reddi AH (1994). Bone and cartilage differentiation. Curr Opin Genet Dev..

[20] Kuo YJ, Wu LC, Sun JS, Chen MH, Sun MG, Tsuang YH (2014). Mechanical stress-induced apoptosis of nucleus pulposus cells: an in vitro and in vivo rat model. J Orthop Sci..

[21] Leckie SK, Bechara BP, Hartman RA, Sowa GA, Woods BI, Coelho JP (2012). Injection of AAV2-BMP2 and AAV2-TIMP1 into the nucleus pulposus slows the course of intervertebral disc degeneration in an in vivo rabbit model. Spine J..

[22] Clouet J, Pot-Vaucel M, Grimandi G, Masson M, Lesoeur J, Fellah BH (2011). Characterization of the age-dependent intervertebral disc changes in rabbit by correlation between MRI, histology and gene expression. BMC Musculoskelet Disord..

[23] Orimo H (2010). The mechanism of mineralization and the role of alkaline phosphatase in health and disease. J Nippon Med Sch..

[24] Kan L, Kessler JA (2014). Evaluation of the cellular origins of heterotopic ossification. Orthopedics..

[25] O'Brien EJ, Frank CB, Shrive NG, Hallgrimsson B, Hart DA (2012). Heterotopic mineralization (ossification or calcification) in tendinopathy or following surgical tendon trauma. Int J Exp Pathol..

[26] Gossl M, Khosla S, Zhang X, Higano N, Jordan KL, Loeffler D (2012). Role of circulating osteogenic progenitor cells in calcific aortic stenosis. J Am Coll Cardiol..

